# 1946. Efficacy of Approved versus Unapproved Vaccines for *SARS-CoV-2* Infection in Randomised Blinded Clinical Trials

**DOI:** 10.1093/ofid/ofac492.1573

**Published:** 2022-12-15

**Authors:** Andrea Perez Navarro, Victoria Pilkington, Andrew Hill, Toby Pepperrell

**Affiliations:** Imperial College London, London, England, United Kingdom; Imperial College London, London, England, United Kingdom; University of Liverpool, London, England, United Kingdom; School of Medicine and Veterinary Medicine, Edinburgh, Edinburgh, Scotland, United Kingdom

## Abstract

**Background:**

Five SARS-CoV-2 vaccines are approved in North America and/or Europe: Pfizer/BioNTech, Moderna, Janssen, Oxford-AstraZeneca and Novavax. Other vaccines have been developed, including Sinopharm, SinoVac, QazVac, Covaxin, Soberana, Corbevac, Medicago, Clover and Cansino, but are not approved in high income countries. This meta-analysis compared the efficacy of approved and unapproved vaccines in randomised clinical trials (RCTs).

**Methods:**

A systematic review of clinical trial registries, PUBMED and EMBASE identified placebo-controlled RCTs of SARS-CoV-2 vaccines prospectively evaluating risks of symptomatic or severe infection with clearly defined endpoints. For each trial, risk of bias was assessed using Cochrane tool 2.0 and the CONSORT checklist. In the pre-defined meta-analysis, relative risks of symptomatic infection and severe disease were compared for each vaccine versus placebo, using Cochrane-Mantel Haenszel Tests (random effects method).

**Results:**

There were 21 RCTs assessing efficacy of the COVID-19 vaccines identified. One RCT was excluded for high risk of bias. Ten RCTs in 206,667 participants evaluated 5 approved vaccines; 10 RCTs in 158,599 participants evaluated 8 unapproved vaccines. In the meta-analysis, prevention of symptomatic infection was 84% (95% C.I. 68-92%) for approved vaccines versus 72% (95% C.I. 65-77%) for unapproved vaccines, with no significant difference between vaccine types (p=0.13). Prevention of severe SARS-CoV-2 infection was 95% (95% C.I. 78-99%) for approved vaccines versus 84% (95% C.I. 72-91%) for unapproved vaccines (p=0.12). In addition, the risk of serious adverse events was similar between vaccine types (p=0.49).

Efficacy of approved and unapproved SARS-CoV-2 vaccines

**Figure:**
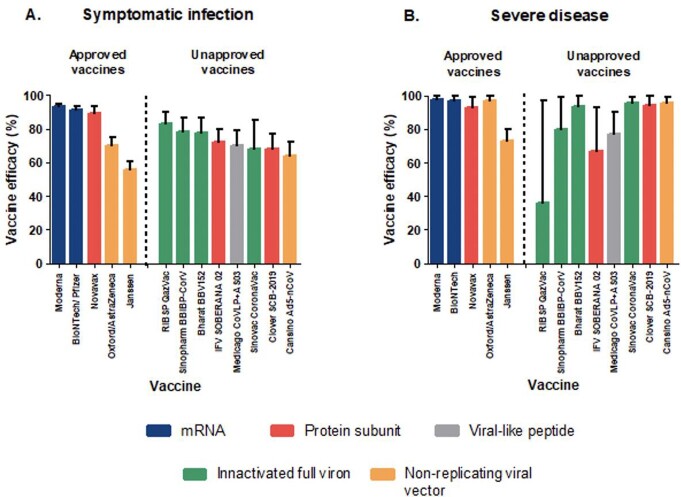

Percentage efficacy of approved and unapproved SARS-CoV-2 vaccines against symptomatic infection (Panel A) and severe disease (Panel B). Vaccines are arranged by approval-status (approved vaccines to the left and unapproved vaccines to the right of discontinuous line) and colour-coded by vaccine type. Error bars represent 95% confidence intervals.

RIBSP, Research Institute for Biological Safety Problems; IFV, Instituto Finlay de Vacunas.

**Conclusion:**

This meta-analysis of 20 RCTs in 365,266 participants, showed no significant difference in efficacy between the approved and unapproved SARS-CoV-2 vaccines for endpoints of either symptomatic or severe infection. Differences in study design, end-point definitions, variants and prevalence of infection may have influenced the results. Head-to-head RCTs will be required to make definitive conclusions. If efficacy is proved definitively, new patent-free vaccines could lower costs of worldwide SARS-CoV-2 vaccination campaigns significantly.

**Disclosures:**

**All Authors**: No reported disclosures.

